# Comprehensive Identification of Human Cell Type Chromatin Activity-Specific and Cell Type Expression-Specific MicroRNAs

**DOI:** 10.3390/ijms23137324

**Published:** 2022-06-30

**Authors:** Yu Han, Yuan Zhou

**Affiliations:** Department of Biomedical Informatics, MOE Key Lab of Cardiovascular Sciences, School of Basic Medical Sciences, Peking University, Beijing 100191, China; sx_hanyu@bjmu.edu.cn

**Keywords:** microRNA, cell type specificity, miRNA expression profiles, chromatin activity, miRNA–disease association

## Abstract

MicroRNAs (miRNAs) regulate multiple transcripts and thus shape the expression landscape of a cell. Information about miRNA expression and distribution across cell types is crucial for the understanding of miRNAs’ functions and their translational applications as biomarkers or therapeutic targets. In this study, we identify cell-type-specific miRNAs by combining multiple correspondence analysis and Gini coefficients to dissect miRNAs’ expression profiles and chromatin activity score profiles, which results in collections of chromatin activity-specific miRNAs in 91 cell types and expression-specific miRNAs in 124 cell types. Moreover, we find that cell-type-specific miRNAs are closely associated with disease miRNAs, such as T-cell-specific miRNAs, which are closely associated with cancer prognosis. Finally, we constructed mirCellType, an online tool based on cell-type-specific miRNA signatures, to dissect the cell type composition of complex samples with miRNA expression profiles.

## 1. Introduction

MicroRNAs (miRNAs) are short, non-coding, single-stranded RNAs that mediate the degradation and/or translational repression of their target mRNAs [1]. As their binding to target mRNAs requires only partial complementary pairing, miRNAs can regulate multiple transcripts in a given cell type [2]. This broad regulatory capacity enables miRNAs to profoundly shape the expression landscape of a cell. In other words, the expression and distribution of miRNA in a cell type are crucial for gene regulation and dysregulation in physiological and pathological conditions, respectively. However, miRNAs are not evenly distributed across cell types, with most having unique expression patterns and some even being expressed only in rare cell populations [3,4,5,6]. In addition, multiple experimental studies have demonstrated that cell-type-specific miRNAs could be associated with a range of human diseases, including cardiovascular diseases, liver diseases, and cancers [7,8]. These lines of experimental evidence also highlight the diagnostic, prognostic, or predictive biomarker capabilities that cell-specific miRNAs may hold. For example, miR-1 and miR-133 are preferentially expressed in cardiac and skeletal muscle cells and can contribute to heart failure by regulating the apoptosis of cardiomyocytes [9,10]. Vascular smooth muscle cell-specific miR-214 exerts a pivotal role in vascular remodeling and hypertension [11]. Hepatocyte-specific miR-122 is a biomarker for drug-induced liver injury (DILI) [12].

Several previous studies have analyzed the cell type specificity of miRNAs in samples with complex cell type composition, such as the blood and nervous system. Juzena et al. utilized differential expression analysis to identify specific miRNA transcriptional signatures in seven blood cells [13]. Jovičić et al. revealed that four principal cell type miRNAs are differentially expressed in the rat cortex to regulate specific phenotypes of neural cell types [14]. However, these studies have investigated only a few abundant cell types in one particular tissue. The lack of attention to less abundant cell types and cell types distributed across multiple tissues limits the accurate identification of the cell type distribution of miRNAs.

With the development of high-throughput small RNA sequencing (sRNA-seq), an extensive atlas of miRNA expression in human cells is being developed: the primary [15,16]. One of the latest advances is that Lorenzi et al. established a miRNA expression atlas across 169 human cell types, providing a foundation for the further exploration of cell type expression-specific miRNAs [15]. However, the samples of these data are cell lines or isolated cells from tissues, resulting in an incomplete range of cell types due to the varying rarity of cell types. Recent single-cell omics technologies should be an excellent supplement to probe the in vivo cell type distribution of miRNAs, but direct single-cell sequencing of miRNAs requires extensive cell processing and, therefore, is still only applicable to a limited number of cells [17,18]. On the contrary, the newly published single-cell chromatin accessibility atlas across 222 cell types [19] has given us an alternative approach. This atlas is established by the sci-ATAC-seq technique, which can address cellular heterogeneity and reveal transcriptionally active regions of individual cells. Thus, the cell type specificity of miRNAs can be explored not only by the expression levels of miRNAs in different cell types, but also by the chromatin accessibility (also called chromatin activity) of miRNA in different cell types.

Here, based on the aforementioned expression and chromatin activity atlases of miRNAs, we identified cell-type-specific miRNAs for 124 and 91 cell types, respectively. We demonstrated the associations of cell-type-specific miRNAs with diseases and cancer prognosis. Finally, we proposed mirCellType, an online tool to infer the cell type composition of complex samples based on the identified cell-type-specific miRNA signatures, which would further facilitate the development of miRNA biomarkers by highlighting the cell-type-specific sources of tumor miRNAs or circulating miRNAs.

## 2. Results

### 2.1. Overview of the Cell-Type-Specific miRNA Catalogs

Based on high-throughput chromatin accessibility and the expression atlas, we obtained a chromatin activity score profile of 1650 miRNAs for 91 cell types and an expression profile of 1198 miRNAs for 124 cell types (see Materials and Methods for details). We next applied the multiple correspondence analysis (MCA) method, a clustering-free multivariate statistical method for the robust extraction of per-cell signatures, to extract the top 20 ranked miRNAs for each cell type in either profile. For each set of the per-cell type miRNA signatures, we only retained miRNAs with a Gini coefficient >0.5 to ensure their absolute specificity. As a result, a miRNA catalog with cell-type-specific expression and a miRNA catalog of cell-type-specific chromatin activity were generated, containing an average of 17.6 and 19.2 cell-type-specific miRNAs per cell type, respectively (Dataset 1 at Figshare: https://doi.org/10.6084/m9.figshare.20186321.v3 (accessed on 19 November 2021), Figure 1A).

**Figure 1:**
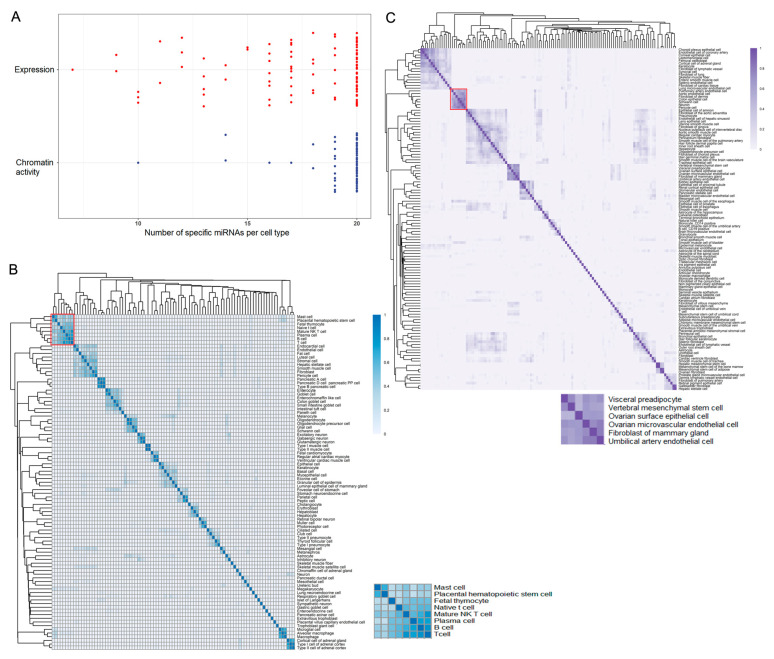
Overview of the cell-type-specific miRNA catalogs. (**A**) Jitter plot showing the number of miRNAs per cell type in the catalog of cell-type-specific miRNAs. (**B**) Clustering heatmap showing the similarities (measured by the Jaccard index) between 91 cell-type-specific chromatin activity miRNA sets. (**C**) Clustering heatmap showing similarities (measured by Jaccard index) between 124 cell-type-specific expression miRNA sets. The 2 representative dense clusters of cell types are highlighted by the red boxes.

A miRNA is not necessarily exclusive to one cell type; instead, similar cell types could share cell-type-specific miRNAs in part. We utilized the Jaccard index, a metric of two-set similarity, to quantify the fraction of specific miRNAs shared by two cell types. The results demonstrate that cell types with similar biological functions or origins had higher similarity in their specific miRNA sets. For instance, the similarity coefficients between the chromatin activity-specific miRNA sets of immune cells such as T cells, B cells, and mast cells were all greater than 0.7 (Figure 1B). In addition, the similarity index between ovarian microvascular endothelial cells and ovarian surface epithelial cells expression-specific miRNA sets was 0.67, suggesting that specific miRNAs are partially shared between similar cell types distributed in the same organ (Figure 1C).

### 2.2. Correlation of Cell Type Specificity of miRNAs with Transcription Factor–miRNA and miRNA–Target Regulations

**Figure 2:**
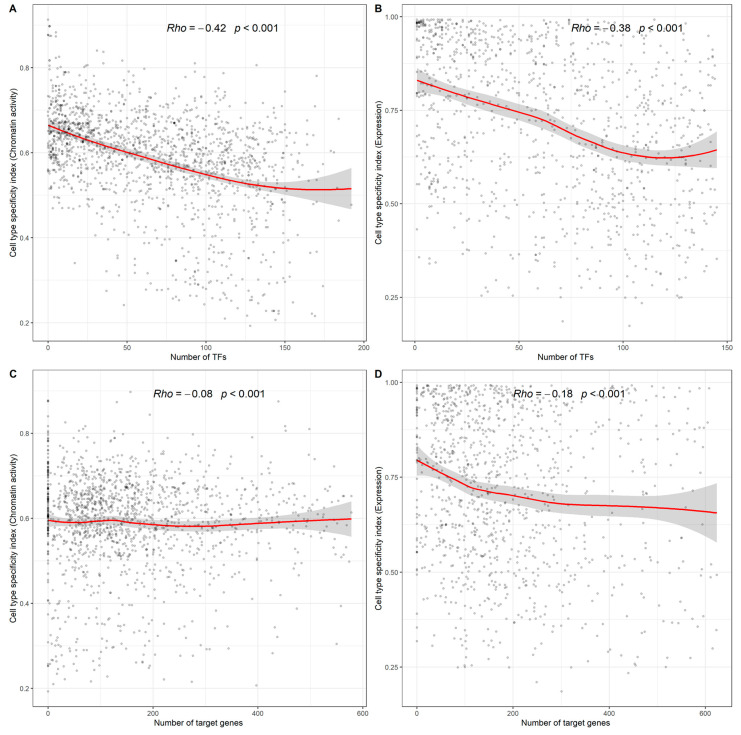
Correlation of cell type specificity of miRNAs with the numbers of regulating TFs and target genes. (**A**) Correlations between cell type chromatin activity specificity index and the number regulating of TFs. (**B**) Correlations between cell type expression specificity index and the number of regulating TFs. (**C**) Correlations between cell type chromatin activity specificity index and the number regulating of target genes. (**D**) Correlations between cell type expression specificity index and the number of regulating target genes.

Transcription factors (TFs) can serve as upstream regulators of miRNAs, activating or repressing miRNA expression. To further understand the regulatory relationship between TFs and cell-type-specific miRNAs, we analyzed the correlation between the cell-type-specific index of a miRNA and the number of TFs that have regulatory associations with the same miRNA. As shown in Figure 2A,B, both the cell type chromatin activity specificity index (Spearman rho = −0.42, *p*-value < 0.001) and the cell type expression specificity index (rho = −0.38, *p*-value < 0.001) were significantly negatively correlated with the number of TFs. That is, the stronger the cell type specificity is, the less TF regulation there is, indicating that extensive TF regulation could be related to wide miRNA expression.

In addition, both the cell type chromatin activity specificity index (rho = −0.08, *p*-value < 0.001) and the cell type expression specificity index (rho = −0.18, *p*-value < 0.001) were also negatively correlated with the number of target genes from the perspective of the downstream regulation of miRNAs. This result suggests that cell-type-specific miRNAs are not extensively involved in the post-transcriptional regulation of genes and tend to play more specific regulatory roles in particular cell types.

### 2.3. Association of Cell-Type-Specific miRNAs with Disease miRNAs

Next, to analyze the relationship between cell-type-specific miRNAs and disease, we investigated the correlation between cell type specificity and disease spectrum width (DSW) [20], which is a metric to assess how extensively a miRNA is involved in human diseases. We only found a negative correlation with DSW for the cell type expression specificity index (rho = −0.21, *p*-value < 0.001, Supplementary Figure S1). Similarly, the expression specificity index had a weak negative correlation with the MIC score [21], an indicator of miRNAs’ importance (rho = −0.16, *p*-value < 0.001, Supplementary Figure S1). The results elucidate that cell-type-specific miRNAs with lower DSW and MIC scores, while not having broad regulatory capacity, are more disease-specific and may show better specificity when adopted as biomarkers.

**Figure 3:**
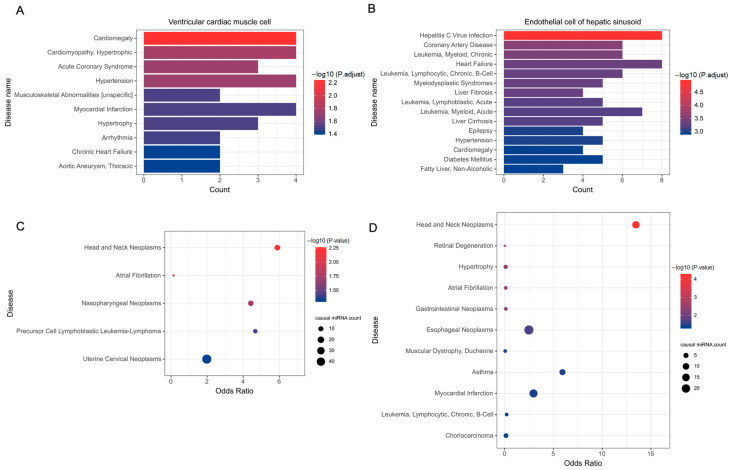
Association of cell-type-specific miRNAs with disease miRNAs. (**A**) Disease association enrichment analysis results of chromatin activity-specific miRNAs in ventricular cardiac muscle cells. (**B**) Disease association enrichment analysis results of expression-specific miRNAs in endothelial cells of the liver sinusoids. (**C**) Associations of cell type chromatin activity-specific miRNAs with disease causal miRNAs. (**D**) Associations of cell type expression-specific miRNAs with disease causal miRNAs.

Moreover, we performed disease association enrichment analysis for each cell-type-specific miRNA set, and obtained significant enrichment in 29 chromatin activity-specific sets and 57 expression-specific sets (Dataset 2: https://doi.org/10.6084/m9.figshare.20186321.v3 (accessed on 19 November 2021)). For example, as shown in Figure 3A, chromatin activity-specific miRNAs in ventricular cardiac muscle cells are enriched in various cardiovascular disease terms. The expression-specific miRNAs in endothelial cells of the liver sinusoids are enriched in liver diseases (e.g., hepatitis C virus infection, and liver fibrosis), cardiovascular diseases (e.g., heart failure and hypertension), and leukemia (Figure 3B).

Furthermore, because disease causal miRNAs are directly involved in disease mechanisms [22,23], we are also concerned with whether there is a difference in cell type specificity between disease causal miRNAs and non-causal miRNAs. The result indicates that causal miRNAs for multiple cancers, such as head and neck tumors, are more inclined to be cell type chromatin activity-specific miRNAs (Figure 3C), while causal miRNAs in asthma and cardiac infarction are more likely to be cell type expression-specific miRNAs (Figure 3D). Together, the above results demonstrate the significant associations between cell-type-specific miRNAs and disease miRNAs, supporting their functional feasibility as potential disease markers.

### 2.4. Relationship between T-Cell-Specific miRNAs and Cancer Prognosis

**Figure 4:**
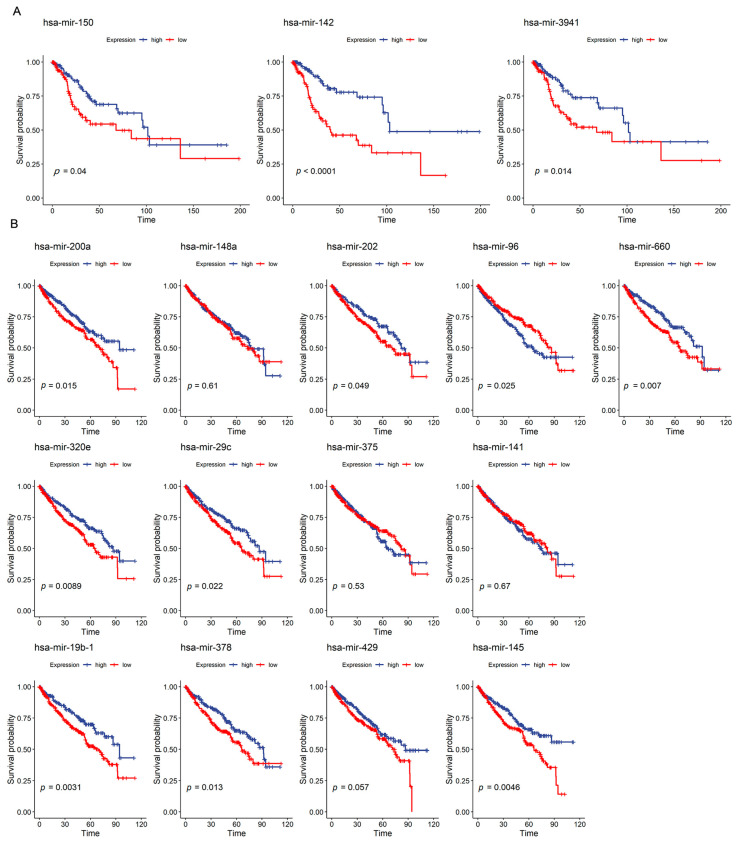
Relationship between T-cell-specific miRNAs and cancer prognosis. (**A**) Survival curves showing the prognostic associations of three T cell chromatin activity-specific miRNAs in CESC. (**B**) Survival curves showing the prognostic associations of thirteen T cell expression-specific miRNAs in KIRC. The red and blue lines indicate miRNAs’ low expression and miRNAs’ high expression, respectively.

Several studies have shown that the dysregulation of individual miRNAs in T cells leads to impaired immune tolerance, which in turn leads to cancer development and progression [24,25]. Moreover, immunotherapy targeting T cells has achieved good outcomes in clinical practice in recent years, so we next focused on the relationship between T-cell-specific miRNAs and cancer prognosis. Using available TCGA clinical data, we determine the impact of T cell chromatin activity-specific miRNAs and expression-specific miRNAs on survival in 35 cancers (Supplementary Figure S2). Although many T cell chromatin activity-specific miRNAs are not included in the TCGA miRNA expression profile, in CESC (cervical squamous cell carcinoma and endocervical adenocarcinoma), higher expression levels of all three T-cell-specific miRNAs with TCGA miRNA expression data (i.e., hsa-mir-150, hsa-mir-142, and hsa-mir-3941) were associated with better prognosis (Figure 4A). Regarding the T cell expression-specific miRNAs, it was observed that in several typical immune-cell-infiltrating tumors, such as BLCA (bladder urothelial carcinoma), HNSC (head and neck squamous cell carcinoma), and KIRC (kidney renal clear cell carcinoma), they are most likely to be associated with cancer prognosis (Supplementary Figure S2A). Indeed, 9 out of the 13 T expression-specific miRNAs affected the survival of KIRC patients, and most of them are beneficial to prognosis (Figure 4B). The expression of T-cell-specific miRNAs is associated with survival and treatment outcomes in different cancers, suggesting that these specific miRNAs may be utilized as prognostic biomarkers in cancer treatments.

### 2.5. mirCellType: An Online Tool to Probe Cell-Type-Specific miRNA Signatures

**Figure 5:**
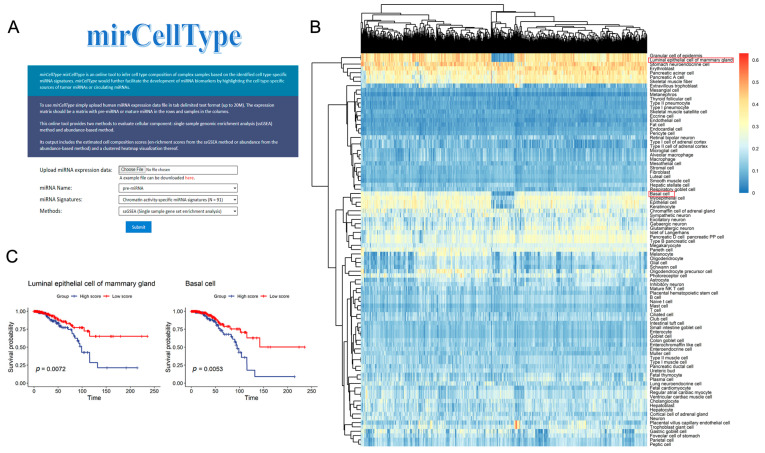
Overview and sample result of the mirCellType online tool. (**A**) The query interface of the mirCellType server. (**B**) Results of mirCellType analysis of BRCA miRNA expression profiles. Enrichment score results are based on cell type chromatin activity-specific miRNAs. (**C**) Kaplan–Meier survival curves showing the distinct survival between patients in the top 25% and bottom 25% enrichment score for luminal epithelial cells of the mammary gland and basal cells.

In cell type heterogeneous complex tissue samples, the average signal of transcriptome measurements is derived from different underlying cell populations. We constructed mirCellType (http://www.rnanut.net/mircelltype/ (accessed on 29 May 2022)), a tool based on cell-type-specific miRNA signatures, to analyze cellular components in complex tissue samples with the miRNA expression profiles as its input. The query interface of mirCellType is shown in Figure 5A. Users can select either miRNA signatures from cell-type-specific expression or those from cell-type-specific chromatin activity. This online tool provides two methods to evaluate cellular components: the single-sample genomic enrichment analysis (ssGSEA) method and the abundance-based method (see Section 4 for details). Its output includes the estimated cell composition scores (enrichment scores from the ssGSEA method or abundance from the abundance-based method) and a clustered heatmap visualization thereof.

To exemplify its usage, we applied mirCellType to the TCGA BRCA (breast invasive carcinoma) miRNA expression profile to map the miRNA landscape of the tumor microenvironment in 1086 samples. We selected cell type chromatin activity-specific miRNA signatures and ssGSEA methods for analysis. In the ssGSEA enrichment score heatmap (Figure 5B), the cellular origin of the miRNAs differed significantly between the tumor samples. We then selected the samples with the top 25% and bottom 25% enrichment scores for luminal epithelial cells of mammary glands or basal cells, and further compared the survival between two groups. Interestingly, patients with low enrichment scores of luminal epithelial cells of mammary glands or low enrichment scores of basal cells have a good prognosis, suggesting an association between the proportion of these two cell types in cancer samples and cancer prognosis (Figure 5C). In addition, we also analyzed BRCA data using abundance-based methods and identified other cell types that might be associated with BRCA prognosis, with scores based on cell type chromatin activity/expression-specific miRNA signatures, and the results are shown in Supplementary Figure S3. These sample results suggest that the mirCellType tool, which applies cell-type-specific miRNA signatures, allows researchers to gain a better insight into the cellular heterogeneity in the cell type mixture of tissue samples.

## 3. Discussion

Cell-type-specific miRNAs play an essential role in shaping cellular identity in health and disease by orchestrating important cellular processes and altering the expression of protein-coding genes [26]. Although many studies have identified cell type expression-specific miRNAs, most studies have investigated only a few cell types in one tissue [13,14]. In recent years, large-scale miRNA expression profiles have been published, mainly in bulk tissue or tumor cell lines [5]. Due to the inherent cellular heterogeneity of tissues, the tissue expression atlas of miRNAs cannot identify the specific cellular origin of miRNA expression. As for cancer cell lines, they are known to have abnormalities in functions and show significant alterations in gene expression profiles compared to normal cells. These issues limit the identification of cell-type-specific expression patterns of miRNAs. It was not until recently that a sizable miRNA expression atlas with various isolated cell types has become available, providing the key resource to identify cell type expression-specific miRNAs [15]. In addition, the single-cell atlas of human genome chromatin accessibility, published just last year [9], provides novel information on cell types because sci-ATAC-seq does not require the isolation of particular cells. It also provides us with a new approach to identify cell-specific miRNAs and to discriminate them from transcriptional activity.

To date, there have been several studies that focused on the cell type specificity of particular tissues. For example, Juzenas et al. proposed a catalog of human peripheral blood cell-specific miRNAs [13], which included only seven cell types. In monocyte cells, we also determined the specificity of hsa-mir-301a, hsa-mir-301b, hsa-mir-301a, and hsa-mir-23a. In agreement with their results, we found that hsa-mir-30a and hsa-mir-577 were specific in B cells. The differences that exist between the two results are likely caused by different miRNA analysis platforms. We also noted some well-known cell-type-specific miRNAs in our results, such as hsa-mir-1, hsa-mir-133a, hsa-mir-133b and hsa-mir-206 in skeletal myofibroblasts and ventricular cardiomyocytes chromatin activity-specific miRNAs. These miRNAs are called myomiRs and can regulate key genes in muscle development and functions [10].

As for the methodology, most of the above studies identified cell-type-specific miRNAs by differential expression analysis. Although this approach is easy to understand, and can quickly find miRNAs with cell type expression pattern restrictions, miRNAs with very low total expression, even with cell type specificity, cannot be identified. Moreover, this approach does not ensure that specific miRNAs are found in all cell types, thus limiting the identification of cell-type-specific miRNAs. In our study, multiple correspondence analysis (MCA), a statistical technique that represents both observations (e.g., cell type) and variables (e.g., miRNAs) in a low-dimensional space, was applied. In MCA biplane plots, analytical distances can be calculated not only between cell types and miRNAs, but also between each cell type and each miRNA to estimate their association. The closer the miRNA is to the cell, the more it can be considered as a specific miRNA for that cell. The most interesting feature of this method is that it allows the determination of the cell-type-specific miRNAs for all cell types in the input miRNA atlas. On this basis, we also set a threshold for the Gini coefficient of each miRNA to ensure that the cell type signature is indeed specific.

In conclusion, we have explored cell-type-specific miRNAs, including chromatin activity-specific and expression-specific miRNAs, in a relatively comprehensive manner. These cell-type-specific miRNA catalogs can be used as a start point to elucidate the role of miRNAs in cell development and specific diseases. On the other hand, our studies have limitations, such as the lack of independent validation with an orthogonal experimental approach. Single-cell small RNA sequencing, although it could be applied to a very limited number of cells in comparison with single-cell mRNA sequencing at the current stage, would be a promising candidate for the validation (and even identification) of cell-type-specific miRNAs in the future.

## 4. Materials and Methods

### 4.1. Data Collection and Processing

Expression profiles of miRNAs across 169 human cell types were taken from Lorenzi et al. [15] and the renormalized counts by library (i.e., counts per million, CPM) were used. As for chromatin activity, we used the peak per cell data from the human genome chromatin accessibility atlas published by Zhang and colleagues [19]. The peak matrix generated from the single-nucleus ATAC-seq data was integrated to form the miRNA chromatin activity matrix, where the chromatin activity of one miRNA in one cell could be quantified by summing the scores of all peaks in the cell within the 2 kb around the genome coordinate of the miRNA. Information on the genome coordinates of miRNA genes was obtained from the miRBase database [26] (https://mirbase.org/, v22, accessed on 19 November 2021). Finally, the cell type chromatin activity was calculated as the average chromatin activity among the cells annotated as the same cell type. To obtain more consistent cell type annotation, all cell type names for both atlases were mapped to the closest Cell Ontology [27] terms, if applicable. By merging and collating the same cell type names after mapping, we finally obtained a chromatin activity profile containing 91 cell types and an expression profile containing 124 cell types.

### 4.2. Cell-Type-Specific miRNA Catalog

We first applied the MCA method to the per cell type expression and chromatin activity matrix of miRNAs to extract the first 20 unbiased miRNA signatures in each cell type. Then, only miRNAs with a Gini coefficient greater than 0.5 were preserved. The retained miRNAs were regarded as cell-type-specific miRNAs.

MCA is a statistical technique that can represent both observations (cell types) and variables (miRNAs) in a low-dimensional space [28,29]. Briefly, we started by linearly converting the expression values in a continuous scale between 0 and 1. MCA performed a dimensionality reduction of the matrix, where both cells and miRNAs were represented in the same vector space. The miRNA ranking of each cell was calculated based on the distance of the miRNAs to the cell in the vector space, where the 20 miRNAs that showed the closest distances were considered as the specific miRNA signatures of the cell type. A detailed description of the MCA method can be found in the original article by Akira et al. [30].

Note that even for cell types that have less specific miRNAs, MCA will still fetch 20 nominal specific miRNAs. Therefore, another hard thresholding of cell type specificity is required. To this end, we calculated the Gini coefficient [31] for each miRNA as the cell-type-specific index. The Gini coefficient can be calculated as follows:

(1) $$\begin{array}{c} Gini=\frac{n+1}{n}-\frac{2\sum_{i=1}^{n}\left(n+1-i\right)x_{i}}{n\sum_{i=1}^{n}x_{i}} \end{array}$$

where *x_i_* is the expression or chromatin activity intensity of the miRNA in the *i*-th cell type (descending ordered). *n* is the number of cell types. The more widespread the distribution of a miRNA is, the closer the value is to 0; the more specific the distribution is, the closer the value is to 1.

### 4.3. Comparative and Correlation Analysis of Cell-Type-Specific miRNA Sets

We first compared specific miRNA signatures from different cell types. Here, we introduced the Jaccard index to explore the similarity of different cell-type-specific miRNAs. The formula is as follows:

(2) $$\begin{array}{c} J\left(A,B\right)=\frac{\left|A\cap B\right|}{\left|A\cup B\right|}=\frac{\left|A\cap B\right|}{\left|A\right|+\left|B\right|-\left|A\cap B\right|} \end{array}$$

where *A* and *B* denote the specific miRNA sets from two different cell types.

To better understand the biological functions of cell-type-specific miRNAs, we performed a series of correlation analyses between the miRNA specificity index and other miRNA biological features. We first downloaded literature-compiled human TF-miRNA regulatory data from TransmiR v2.0 (http://www.cuilab.cn/transmir, accessed on 5 May 2021) [32] and counted the number of transcription factors regulating each cell-type-specific miRNA. Similarly, miRNA–mRNA targeting relationship data supported by experimental evidence were downloaded from miRTarBase v8.0 (http://miRTarBase.cuhk.edu.cn/, accessed on 5 May 2021) [33] and the number of target genes of each cell-type-specific miRNA was counted. In addition, Disease Spectrum Width (DSW) scores were calculated using human miRNA–disease associations from the HMDD v3.2 database (http://www.cuilab.cn/hmdd/, accessed on 19 November 2021) [22], which represent the importance of a miRNA in human diseases. We also obtained MicroRNA Importance Calculator (MIC) scores [21] as another measure of the importance of miRNA from http://www.cuilab.cn/mic/ (accessed on 19 November 2021).

### 4.4. Disease Association Enrichment Analysis

The whole set of experimentally validated miRNA-disease associations and the causal miRNA-disease association subset were obtained from HMDD v3.2 (http://www.cuilab.cn/hmdd/, accessed on 19 November 2021) [22]. The enrichment of disease associated miRNAs among cell-type-specific miRNAs were analyzed by the clusterProfiler R functional enrichment package with customized annotation files recording the known associations between miRNAs and diseases [34]. FDR < 0.05 was defined as the threshold of significance. We further focused on miRNAs that are causally associated with diseases, which are generally directly involved in disease mechanisms [22,23]. The Fisher’s exact test was used to determine whether there is a significant association between the causal miRNAs of one disease and the specific miRNAs of one cell type. Only diseases containing at least 5 causal association miRNAs were considered here to avoid less confident results from poorly annotated diseases.

### 4.5. TCGA Data Collection and Survival Analysis

MiRNA expression data in tumor samples were retrieved from the TCGA database (https://portal.gdc.cancer.gov/, accessed on 15 April 2022). Samples from patients without any available survival time or events were removed. We obtained data for 35 cancer types: ACC, BLCA, BRCA, CESC, CHOL, COAD, DLBC, ESCA, FPPP, HNSC, KICH, KIPAN, KIRC, KIRP, LAML, LGG, LIHC, LUAD, LUSC, MESO, OV, PAAD, PCPG, PRAD, READ, SARC, SKCM, STAD, STES, TGCT, THCA, THYM, UCEC, UCS, and UVM. For each cancer type, we performed survival analysis for T cell chromatin activity-specific miRNAs and T cell expression-specific miRNAs, based on the TCGA miRNA expression and survival information. Survival curves were generated using the Kaplan–Meier method. Based on the expression of each miRNA, each sample was assigned to one of two groups: a low-expression group or a high-expression group. The log-rank test was used to assess the statistical significance of the survival differences between the high- and low-expression categories. The significance cut-off was *p* < 0.05. Kaplan–Meier analysis and log-rank test were performed using the R package survival.

### 4.6. mirCellType Server Construction

We established mirCellType, an online tool to infer the cell type composition of complex/bulk samples (e.g., peripherical blood) based on the identified cell-type-specific miRNA signatures. Its input was a miRNA expression matrix, where rows were miRNAs and columns were samples. Repeated miRNA names were combined. mirCellType provided two analysis methods. The first one adopted ssGSEA (single-sample genomic enrichment analysis) to score each sample. ssGSEA determined an enrichment score of a set of cell-type-specific miRNAs in the top of a ranked miRNA expression profile. The ssGSEA algorithm was implemented by using the R package GSVA. The second method was to calculate the expression abundance of specific miRNAs. We calculated the corresponding per-sample scores, which were also called abundance scores here, by using the log2 geometric mean of cell-type-specific miRNAs. The web interface and webserver construction were accomplished with the HTML + PHP + Apache framework.

## Data Availability

The mirCellType online tool is available at http://www.rnanut.net/mircelltype/ (accessed on 29 May 2022). The cell type-specific miRNA catalogs (Dataset 1) and disease association enrichment analysis results (Dataset 2) presented in this study are openly available in FigShare at https://doi.org/10.6084/m9.figshare.20186321.v3 (accessed on 19 November 2021).

## Supplementary Materials

The following supporting information can be downloaded at: https://www.mdpi.com/article/10.3390/ijms23137324/s1.
