# Supplementary material for: Comprehensive Identification of Human Cell Type Chromatin Activity-Specific and Cell Type Expression-Specific MicroRNAs
Source: Int J Mol Sci. 2022 Jun 30;23(13):7324. doi: 10.3390/ijms23137324 (PMC9266980; doi:10.3390/ijms23137324)
Supplement: Supplementary file 1 [file ijms-23-07324-s001.zip › ijms-1770799-supplementary.pdf]

Supplementary Materials

Comprehensive identification of human cell type chromatin activity-specific and cell type expression-specific microRNAs

Supplementary Figures

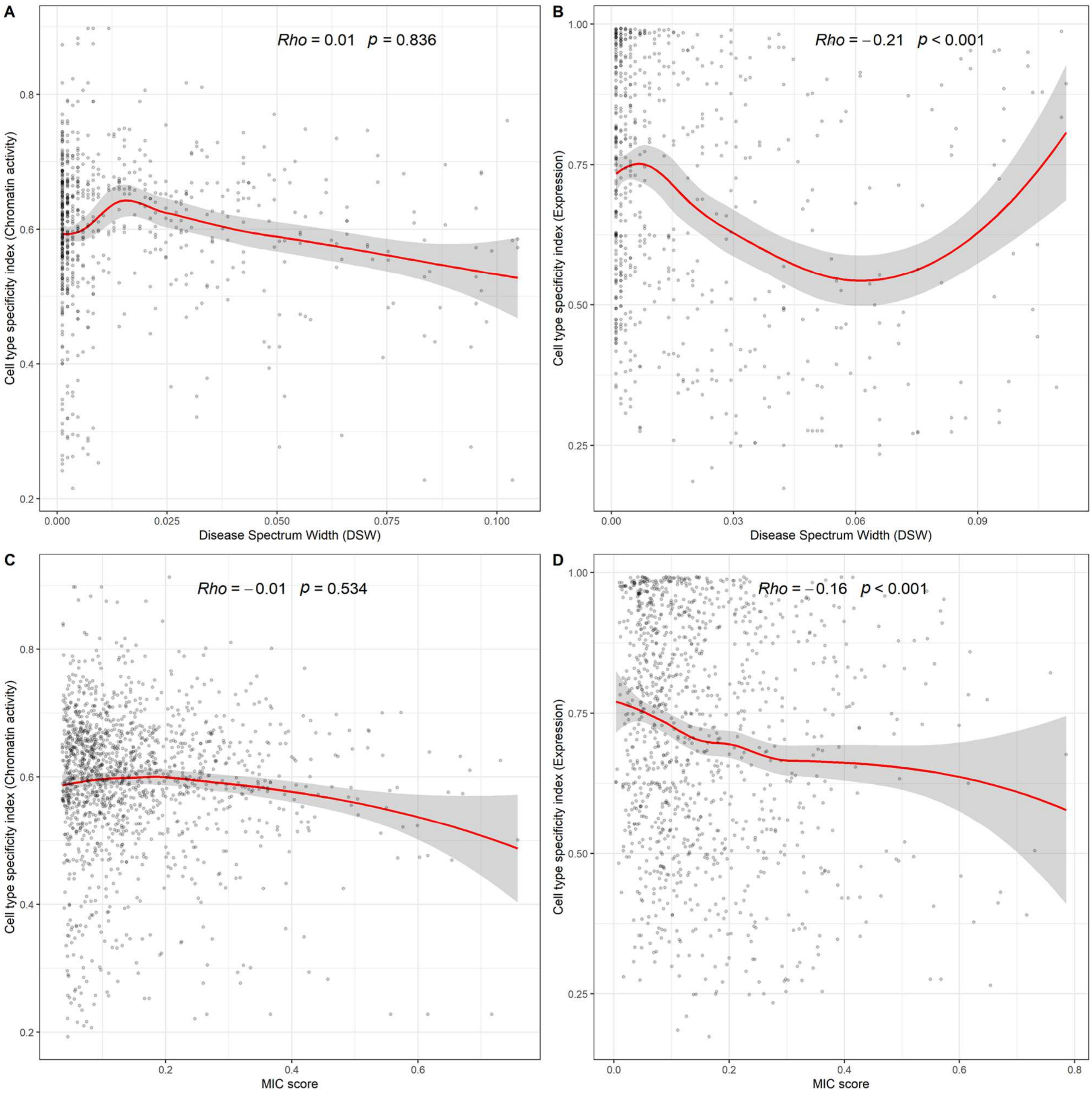

Supplementary Figure S1. **Correlation of cell-type specificity of miRNAs with DSW and MIC score.** (A) Correlations between cell type chromatin activity specificity index and DSW score. (B) Correlations between cell type expression specificity index and DSW score. (C) Correlations between cell type chromatin activity specificity index and MIC score. (D) Correlations between cell type expression specificity index and MIC score.

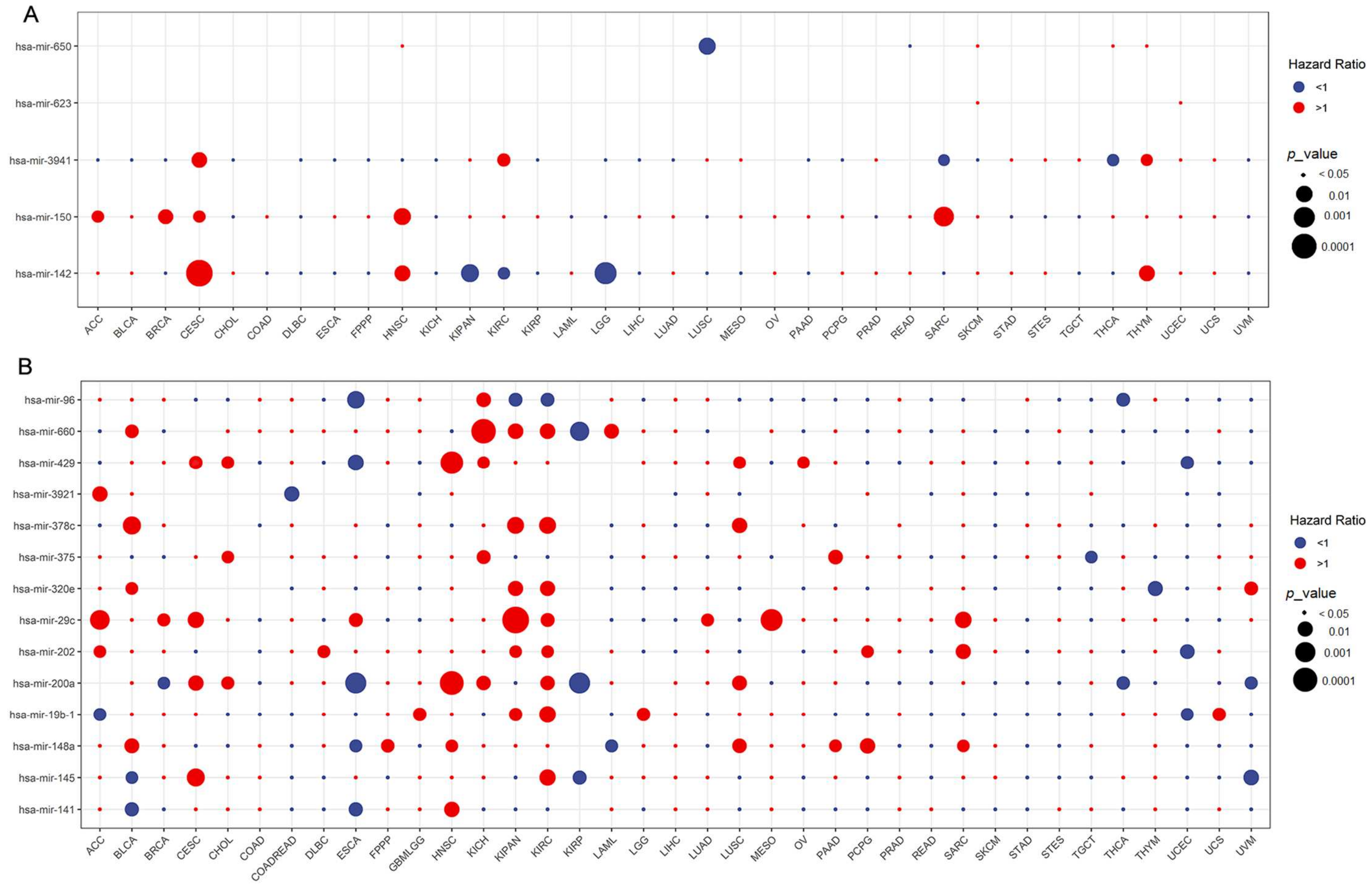

Supplementary Figure S2. Relationship between T cell-specific miRNAs and cancer prognosis.
(A) Survival analysis of high and low T cell chromatin activity-specific miRNAs expression on the prognosis of TCGA cancers patients. (B) Survival analysis of high and low T cell expression-specific miRNAs expression on the prognosis of TCGA cancers patients. The circle size represents the log-rank test *p*-value, Hazard Ratio>1 color is blue, >1 is red. Grid blanks represent missing data for the corresponding miRNAs in the cancer data.

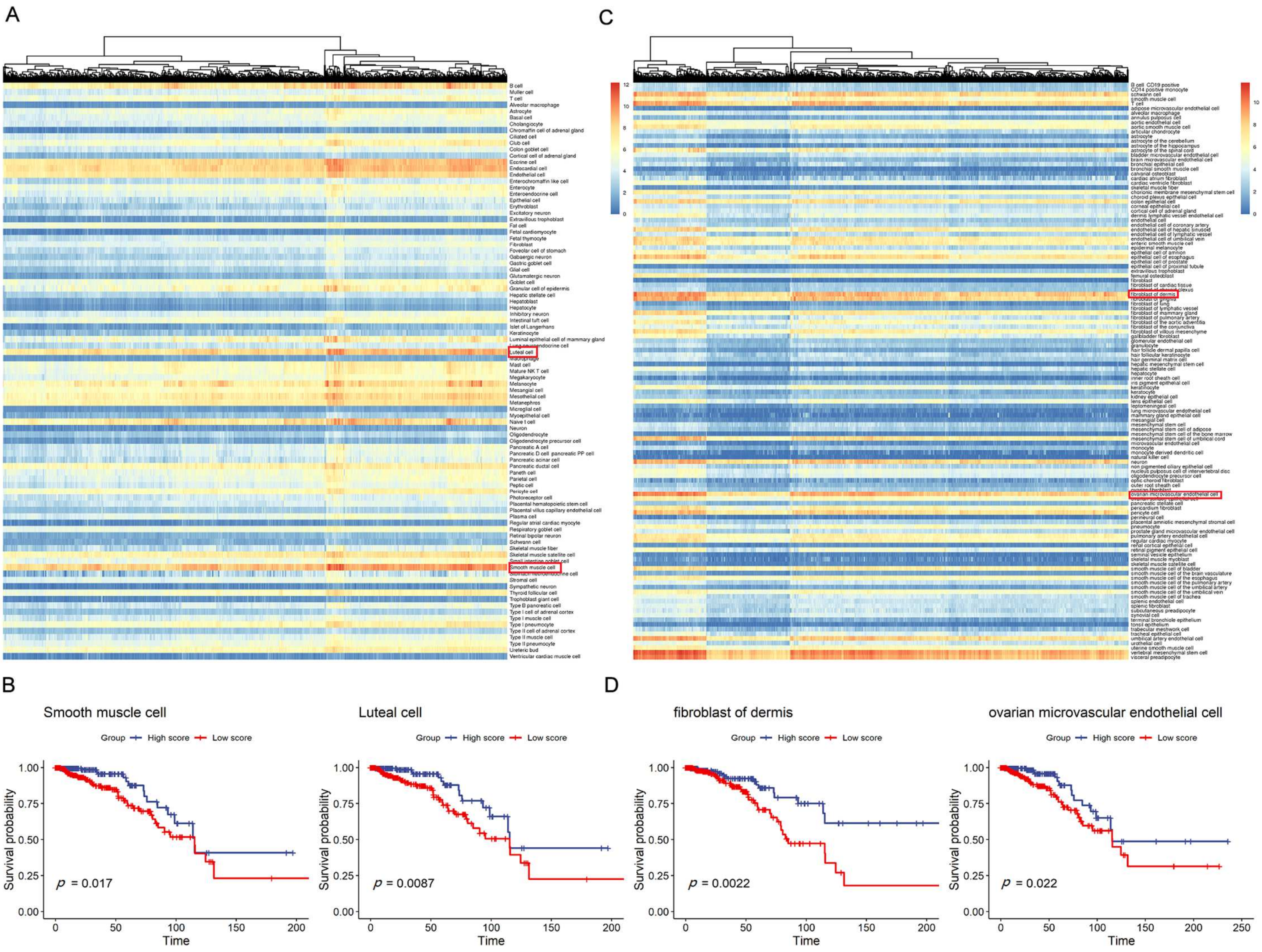

Supplementary Figure S3. Sample result of the mirCellType online tool.

(A) Results of mirCellType analysis of BRCA miRNA expression profiles using abundance-based method. Abundance results are based on cell type chromatin activity-specific miRNAs. (B) Kaplan–Meier survival curves showing the distinct survival between patients in the top 25% and bottom 25% abundance for smooth muscle cell and luteal cell. (C) Results of mirCellType analysis of BRCA miRNA expression profiles using abundance-based method. Abundance results are based on cell type expression-specific miRNAs. (D) Kaplan–Meier survival curves showing the distinct survival between patients in the top 25% and bottom 25% abundance for fibroblast of dermis and ovarian microvascular endothelial cell.
